# Effects of Young Barley Leaf Powder on Gastrointestinal Functions in Rats and Its Efficacy-Related Physicochemical Properties

**DOI:** 10.1155/2014/974840

**Published:** 2014-07-08

**Authors:** Motoya Ikeguchi, Masahito Tsubata, Akira Takano, Tomoyasu Kamiya, Kinya Takagaki, Hideyuki Ito, Yohko Sugawa-Katayama, Hideaki Tsuji

**Affiliations:** ^1^Research and Development Division, Toyo Shinyaku Co. Ltd., 7-28 Yayoigaoka, Tosu-shi, Saga 841-0005, Japan; ^2^Faculty of Health and Welfare Science, Okayama Prefectural University, 111 Kuboki, Soja-shi, Okayama 719-1197, Japan; ^3^Department of Health and Nutrition, Osaka Aoyama University, 2-11-1 Niina, Minoh-shi, Osaka 562-8580, Japan

## Abstract

Young barley leaf is consumed as a popular green-colored drink, which is named “Aojiru” in Japan. In the present study, we examined effects of young barley leaf powder (BL) on gastrointestinal transit time (GTT) and fecal moisture and weight in comparison with wheat bran (WB) in male Sprague-Dawley rats. In addition, an attempt was made to identify BL components responsible for these effects by using various fractions of BL. Additionally, we examined the water-holding capacity and setting volume of BL *in vitro*. We also examined the granular structures of BL with a scanning electron microscope. As a result, BL supplementation in the diet increased the fecal weight and shortened GTT. Our results demonstrate that the active component responsible for the effect on increasing the fecal volume in BL is the water-insoluble dietary fiber fraction and that this effect is thought to be caused by stimulation of the gut tract by the pH lowering. Furthermore, the high laxative action of BL was thought to be ascribable to the high water-holding capacity due to the complicated structures of BL.

## 1. Introduction

Barley (*Hordeum vulgare* L.) is a plant widely distributed and cultivated in eastern Asia and used as foodstuffs such as bread and cakes [1–3]. Young barley leaf is well-known as the material of a green-colored drink named “Aojiru” in Japan [3]. Recently, several animal and human studies revealed that the young barley leaves exert beneficial effects such as antiulcer, antioxidant, hypolipidemic, antidepressant, and antidiabetic effects [4–8]. Moreover, we previously reported effect of young barley leaf powder (BL) on gastrointestinal functions of human healthy volunteers and those with mild constipation [9–11]. In these studies, we found that 2 g to 10 g of BL supplementation increased fecal weight and defecation frequency and improved other fecal characteristics. BL is rich in insoluble dietary fiber. The effects of insoluble dietary fiber on gastrointestinal functions have been described in many reports [12–15]. However, BL also possesses water-soluble dietary fiber, and some kinds of soluble dietary fiber are also known to have effects on the gastrointestinal functions [16, 17]. In the present study, we examined the effects of BL on gastrointestinal transit time (GTT) and the moisture and weight of feces compared with wheat bran (WB) in male Sprague-Dawley (SD) rats. In addition, an attempt was made to identify the BL component responsible for these effects by using various fractions of BL. Additionally, we examined the water-holding capacity (WHC) and setting volume in water (SV) of BL* in vitro*. We also examined the granular structures of BL with a scanning electron microscope.

## 2. Materials and Methods

### 2.1. Materials

**Figure 1:**
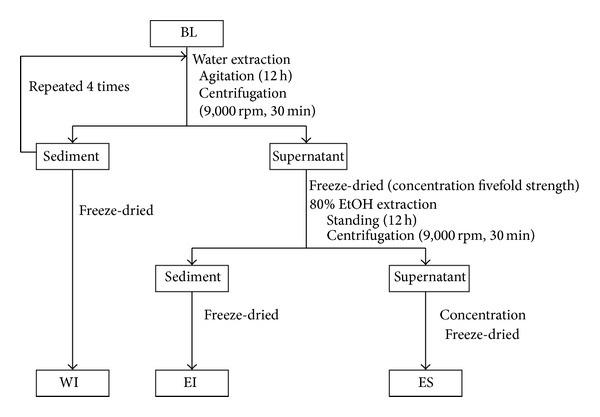
The preparation method of fractions of young barley leaf powder. BL: young barley leaf powder, WI: water-insoluble fraction of BL, EI: ethanol-insoluble fraction of BL, and ES: ethanol-soluble fraction of BL.

BL was supplied by Toyo Shinyaku Co., Ltd. (Saga, Japan). BL is produced from the young leaves of* Hordeum vulgare* L. by washing, drying, and powdering. The color of BL ranges from light to dark green. The whole of BL was separated into three fractions: water-insoluble fraction (WI), ethanol-insoluble fraction (EI), and ethanol-soluble fraction (ES).  Figure 1 shows the scheme of fractionation. Briefly, BL suspended in water was agitated for 12 hours and centrifuged for 30 minutes at 9,000 rpm. After this process was repeated 4 times, BL was separated into the supernatant and the sediment. The sediment was freeze-dried (WI). The supernatant was concentrated fivefold by freezing and drying. After adding four volumes of 80% ethanol, the mixture was left to stand overnight. The mixture was centrifuged for 30 minutes at 9,000 rpm and separated into two fractions: EI and ES. The percentages by weight of WI, EI, and ES were 82.51%, 6.32%, and 13.53%, respectively, to the weight of BL. WB (a trade name; Wheat bran P) was supplied by Nisshin Seifun Co., Ltd. (Tokyo, Japan).  Table 1 shows the chemical compositions of BL, WI, EI, ES, and WB.

### 2.2. Animal Experiments

Animal experiments in the present study were approved by the Ethical Committee of TOYO SHINYAKU Co., Ltd. All study designs complied with the Guidelines for the Care and Use of Experimental Animals of the Japanese Association for Laboratory Animal Science.

*(1) Animal-Rearing Conditions and Diet Compositions*. Thirty-six male SD rats were purchased from Japan SLC Inc. (Shizuoka, Japan) at the age of 5 weeks. They were housed in a room with controlled temperature (23 ± 2°C), humidity (55 ± 10%), and a preset light-dark cycle (12 h : 12 h). The rats were fed laboratory chow (MF, Oriental Yeast Co., Ltd., Tokyo, Japan) for 1 week during the acclimatization period, and then the 36 rats were randomly divided into 6 groups with approximately equal average body weights. The animals were housed individually in cages and allowed free access to powdered diets and drinking water for the experimental period of 28 days. In order to examine alternation of cecum pH, the animals were treated with BL for 28 days as previously reported [18].  Table 2 shows the diet compositions. The control (CT) group was fed a modified AIN- (American Institute of Nutrition-) 76 diet in which the percentage of cellulose was decreased from 5% to 3%. The WB and BL groups were fed supplemented diets in which sucrose was partly replaced by test materials, WB (5.25%) and BL (3.51%), respectively, so that each diet contained a total of 5% dietary fiber based on the fiber contents of WB (38.1%) and BL (57.0%) shown in Table 1. The diets for the WI, EI, and ES groups contained each fraction in amounts corresponding to the respective contents 82.51%, 6.32%, and 13.53% in BL (see above). The animal weight and feed consumption were measured every 3 or 4 days.

*(2) Gastrointestinal Transit Time (GTT)*. GTT was measured on days 19 and 20 of the feeding period. After 9 hours fasting, the animals were given 2 g of each diet containing 0.5% red pigment (Carmin; Wako Pure Chemical Industries, Ltd. (Osaka, Japan)) between 21:00 and 22:00. The animals were then allowed free access to each experimental diet. We observed fecal conditions every hour after the stained diet. GTT was calculated as the average time between the appearance and disappearance of stained feces.

*(3) Measurement of Fecal Moisture Contents and Weights*. Fecal moisture was measured on days 11 and 18 of the feeding experiment. Briefly, two pieces of fresh feces were collected directly from the anus with a test tube and their weights were measured quickly and dried overnight at 100°C in an incubator. Fecal moisture contents were calculated as the difference between the fresh and dried feces.

In order to measure dry fecal weights, we collected all excreted feces continuously for 4 days (from day 25 to 28 of the feeding period). At 10:00 every day, we collected all feces excreted during the preceding 24 hours. All the collected feces were dried overnight at 100°C in the incubator and their weights were measured as dry fecal weights.

*(4) Wet Weight and Length of the Colon and pH of the Cecal Contents*. On the final day (day 28) of the experiment, the animals were anesthetized with ether. After the animals were sacrificed by exsanguination, laparotomy was performed. The small intestine, cecum, and colon were excised and their lengths and weights were measured. The small and large intestines were removed and irrigated with saline. After dabbing away the excess water, the small and large intestines were weighed. The cecal contents were diluted tenfold with distillated water, and their pH values were measured by the glass electrode method.

### 2.3. Materials for* In Vitro* Experiments

BL and WB were the same samples as used in the above animal study. Cellulose (CL; a trade name; S-102) was supplied by Takeda Pharmaceutical Co., Ltd. (Tokyo, Japan). The contents of dietary fiber in BL, WB, and CL were 37.8%, 38.1%, and 96.0%, respectively.

### 2.4. *In Vitro* Experiments

*(1) Water-Holding Capacity (WHC) [19]*. We used a polyethylene container with a mesh wire-bottom of stainless steel (6 cm in diameter and 8 cm in depth) with fitted filter paper in the bottom. First, the filter in the container was moisturized adequately by water and weighed every 2 minutes. When the speed of loss of weight began to become constant, the weight was determined as W1. Secondly, each dried one gram sample of BL, WB, and CL was shaken intermittently with 75 mL of water in a beaker and poured on the filter in the container. Each sample in the container was weighed every 2 minutes. When the speed of loss of weight began to become constant, the weight was determined as W2. WHC was obtained by the calculation formula: W2 − W1 − 1 gram.

*(2) Setting Volume in Water (SV) [19]*. The method was developed by Middleton and Byers [20] and applied by Takeda et al. for the determination of SV of dietary fiber. Briefly, each dried 5 g sample of BL, WB, and CL was shaken intermittently with 50 mL of deionized water placed in a 200 mL media bottle under reduced pressure. Each suspension was transferred to a 100 mL graduated cylinder and left to stand quietly for 24 hours. SV was measured as the height of sedimentation of each sample in the cylinder.

### 2.5. Photographs with a Scanning Electron Microscope (SEM)

For taking photographs of expanded images of BL, WB, and CL, we used SEM (MINISCOPE TM-1000, Hitachi High-Technologies Co., Ltd.). We observed each sample at 100- to 500-fold magnification on the SEM.

### 2.6. Statistics

Data were expressed as means ± SEM. Statistical analyses were done with one-way ANOVA to compare all subgroups and the Tukey test for paired comparisons. In all experiments, significance was set at *P* < 0.05.

## 3. Results

### 3.1. Animal Experiments

*(1) Weights and Feed Consumptions*. There were no significant difference in body weights and feed consumptions (data not shown).

**Figure 2:**
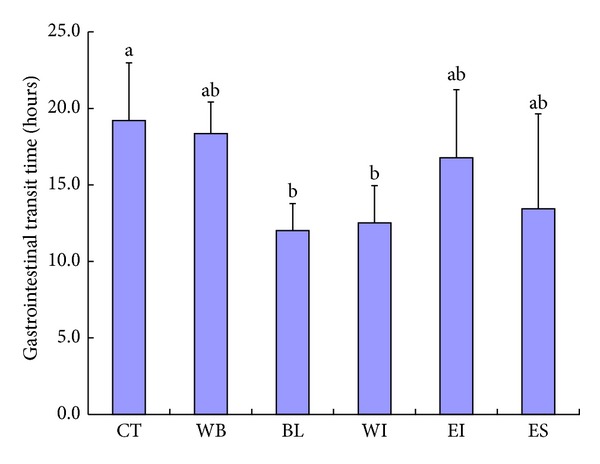
Gastrointestinal transit time in rats. CT: control group, WB: wheat bran group, BL: whole of young barley leaf powder (BL) group, WI: water-insoluble fraction of BL group, EI: ethanol-insoluble fraction of BL group, and ES: ethanol-soluble fraction of BL group. a and b Data with different alphabetical letters are significantly different at *P* < 0.05.

*(2) Gastrointestinal Transit Times*.  Figure 2 shows the result of GTT measurement. The GTTs of the BL and WI groups were shortened compared with that of the CT group. On the other hand, the GTTs of the WB, EI, and ES groups were not different from the GTT of the CT group.

**Figure 3:**
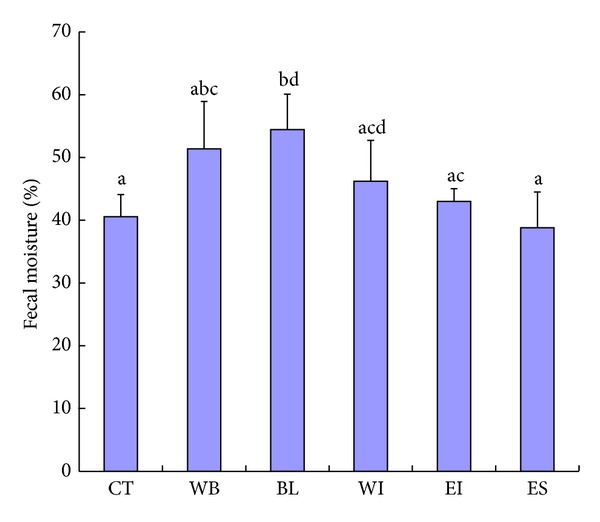
Fecal moisture in rats. CT: control group, WB: wheat bran group, BL: whole of young barley leaf powder (BL) group, WI: water-insoluble fraction of BL group, EI: ethanol-insoluble fraction of BL group, and ES: ethanol-soluble fraction of BL group. a–d Data with different alphabetical letters are significantly different at *P* < 0.05.

**Figure 4:**
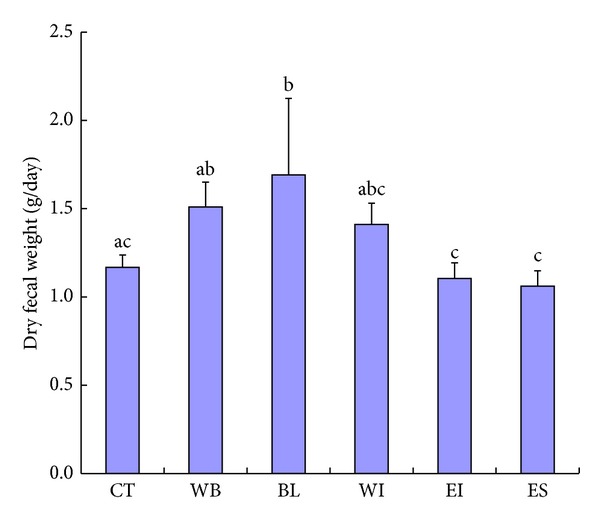
Dry fecal weight in rats. CT: control group, WB: wheat bran group, BL: whole of young barley leaf powder (BL) group, WI: water-insoluble fraction of BL group, EI: ethanol-insoluble fraction of BL group, and ES: ethanol-soluble fraction of BL group. a–c: Data with different alphabetical letters are significantly different at *P* < 0.05.

*(3) Fecal Moisture Contents and Weights*. Figures 3 and 4 show the results of fecal moisture contents and dry fecal weight. The fecal moisture content of the BL group was higher than that of the CT group. The fecal moistures contents of the WB, WI, and EI groups tended to be higher than that of the CT group. On the other hand, the fecal moisture content of the ES group was similar to that of the CT group. The dry fecal weight of the BL group increased compared with that of the CT group. The dry fecal weights of the WB and WI groups tended to increase compared with that of the CT group. On the other hand, the dry fecal weights of the EI and ES groups were similar to that of the CT group.

*(4) Wet Weight and Length of the Colon and pH of the Cecal Contents*. Among the wet weights and tissue weights of the small intestine, cecum, and colon, there were no significant differences in any of the groups. In addition, there were no significant difference in the lengths of small and large intestines (data not shown).  Table 3 shows the result of pH of the cecal contents. The pH values of the cecal contents of the WB, BL, and WI groups were significantly lower than that of the CT group. On the other hand, the pH values of the EI and ES groups were similar to that of the CT group.

### 3.2. *In Vitro* Experiments

Table 4 shows the results of WHC and SV measurement. The WHC and SV of BL were higher than those of WB and CL. The WHC and SV of WB were similar to those of CL.

### 3.3. Photographs with a Scanning Electron Microscope (SEM)

**Figure 5:**
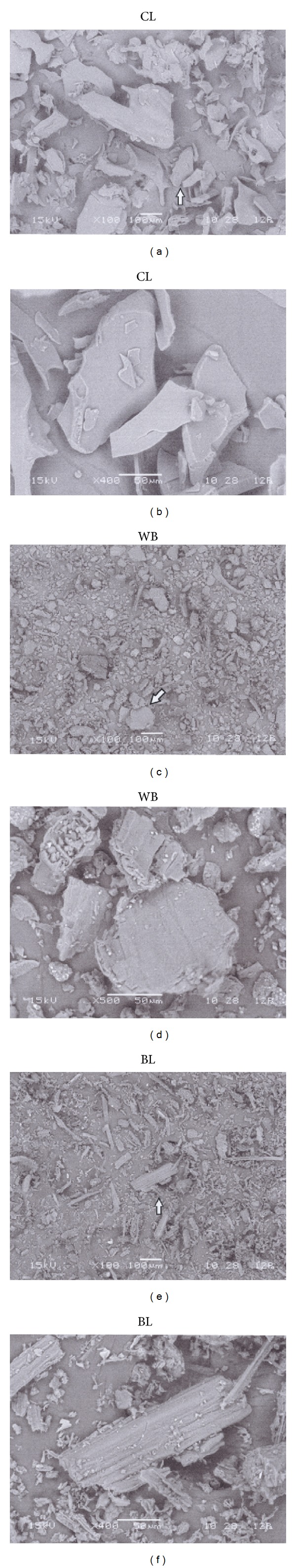
Photographs of cellulose, wheat bran, and young barley leaf powder with scanning electron microscope. (a) cellulose powder (100-fold magnification), (b) cellulose powder (400-fold magnification), (c) wheat bran powder (100-fold magnification), (d) wheat bran powder (500-fold magnification), (e) young barley leaf powder (100-fold magnification), and (f) young barley leaf powder (400-fold magnification).

Figure 5 shows the SEM images of BL, WB, and CL. BL and WB are suggested to have many fine grains which have small holes. In addition, the large grains of BL have many fine slits on the surfaces and seem to have complicated structures. On the other hand, WB and CL have no slits and seem to have very smooth surfaces.

## 4. Discussion

When we surveyed the effect of WB, BL, and each fraction of BL in this animal study, the dry fecal weight of the BL group increased and that of the WB or WI group tended to increase compared with that of the CT group, but those of the EI and ES groups were similar to that of the CT group. WB and BL contained high amounts of dietary fiber. Moreover, when WI was prepared, the ratio of water-insoluble dietary fiber increased. It is well-known that insoluble dietary fiber is hard to disintegrate by digestive fluids and enzymes in the upper gut and tends to reach the colon as structurally intact molecules, and they are used to form the building frame of feces [21]. Therefore, the effects of BL to increase the fecal volume should be ascribable to the insoluble dietary fiber of BL.

Additionally, It is known that some kinds of dietary fiber (both soluble and insoluble fibers) and oligosaccharides are utilized partially by intestinal flora, and short-chain fatty acids produced by the flora cause a lowering of pH, which stimulates the gut tract and promotes the elimination of feces [13, 22–25]. In this animal study, pH of the cecal contents of the BL and WI group was lowered. Previously, we found that treatment of female volunteers with BL caused a reduced percentage of Clostridium (so-called “bad bacterium”) in their feces [9]. Short-chain fatty acids were able to inhibit pathogenic bacterial growth by lowering pH in the intestinal lumen [26]. The insoluble dietary fiber of BL might cause an increase in the fermentative activity of some kinds of intestinal flora and promote fermentation of intestinal contents and improve the amount of defecation.

On the other hand, the GTT of the BL or WI group was shortened compared with that of the CT group. However, the GTT of the EI or ES group was not shortened. Additionally, despite the fact that WB has high amount of dietary fiber and tends to increase the dry fecal weight and decrease cecal pH, the GTT of WB group was not shortened compared with that of the CT group. Therefore, the effect of BL to shorten GTT could not be explained only by the high amount of insoluble dietary fiber.

When we surveyed the effects of water-holding capacity of BL, WB, and CL in the* in vitro* study, the augmenting effects of BL on WHC and SV were greater than those of WB or CL. The SEM observation of BL, WB, and CL showed that BL has more complicated structures than WB or CL. In the study using inert plastic particles of different sizes and shapes, a coarse-grained plastic had complicated structures and showed a greater effect on SV than a fine-grained one, and the coarse-grained plastic caused shortening of GTT compared with the fine-grained one [27]. The shortening effect of BL on GTT was thought to be ascribable to the high water-holding capacity due to the complicated structures of BL.

In conclusion, BL supplementation in the diet increased the fecal weight and shortened the GTT. Our results suggest that the active component in BL responsible for the augmenting effects on the intestinal transit and fecal volume is likely to be the insoluble dietary fiber fraction and that these effects are thought to be caused by the presence of insoluble dietary fiber as the building frame of feces and stimulation of the gut tract by the pH lowering. Furthermore, the high laxative action of BL was thought to be ascribable to the high water-holding capacity due to the complicated structures of BL.

## Figures and Tables

**Table 1:** Chemical compositions of samples in the animal experiments.

	Unit	WB	BL	WI	EI	ES
Energy^∗1^	kcal	299	262	—	—	—
Moisture	g	3.5	4.9	—	—	—
Protein	g	24.3	14.7	—	—	—
Fat	g	2.6	4.2	—	—	—
Ash	g	6.0	6.3	—	—	—
Carbohydrate	g	25.5	12.9	—	—	—
Dietary fiber^∗2^	g	38.1	57.0	—	—	—

Dietary fiber^∗3^	g	35.8	57.6	72.9	15.1	1.5
Soluble dietary fiber^∗4^	g	3.6	1.6	1.4	13.8	1.2
Hemicellulose^∗4^	g	21.1	23.0	28.4	1.3	0.3
Cellulose^∗4^	g	8.2	26.7	33	ND	ND
Lignin^∗4^	g	2.9	6.3	10.1	ND	ND

^∗1^Conversion factor into calorie (protein 4, fat 9, carbohydrate 4, and dietary fiber 2).

^∗2^Enzymatic-gravimetric method.

^∗3^Southgate method.

^∗4^Phenol-sulfuric acid method.

WB: wheat bran, BL: whole of young barley leaf powder (BL), WI: water-insoluble fraction of BL, EI: ethanol-insoluble fraction of BL, and ES: ethanol-soluble fraction of BL.

**Table 2:** Diet compositions of the animal experiments.

	(%)					
	CT group	WB group	BL group	WI group	EI group	ES group
Casein	20.00	20.00	20.00	20.00	20.00	20.00
DL-Methionine	0.30	0.30	0.30	0.30	0.30	0.30
Cornstarch	15.00	15.00	15.00	15.00	15.00	15.00
Sucrose	52.00	46.75	48.49	49.10	51.78	51.53
Corn oil	5.00	5.00	5.00	5.00	5.00	5.00
AIN mineral mixture	3.50	3.50	3.50	3.50	3.50	3.50
AIN vitamin mixture	1.00	1.00	1.00	1.00	1.00	1.00
Choline bitartrate	0.20	0.20	0.20	0.20	0.20	0.20
Cellulose powder	3.00	3.00	3.00	3.00	3.00	3.00
WB	—	5.25	—	—	—	—
WH	—	—	3.51	—	—	—
WI	—	—	—	2.90	—	—
EI	—	—	—	—	0.22	—
ES	—	—	—	—	—	0.47

CT: control, WB; wheat bran, BL: whole of young barley leaf powder (BL), WI: water-insoluble fraction of BL, EI: ethanol-insoluble fraction of BL, and ES: ethanol-soluble fraction of BL.

**Table 3:** The pH of cecal contents.

	pH
CT group	7.08 ± 0.13^a^
WB group	6.87 ± 0.17^b^
BL group	6.87 ± 0.11^b^
WI group	6.75 ± 0.07^b^
EI group	7.24 ± 0.09^a^
ES group	7.10 ± 0.12^a^

CT: control, WB: wheat bran, BL: whole of young barley leaf powder (BL), WI: water-insoluble fraction of BL, EI: ethanol-insoluble fraction of BL, and ES: ethanol-soluble fraction of BL.

^a,b^Data with different alphabetical letters are significantly different at *P* < 0.05.

**Table 4:** Water-holding capacity and setting volume in water of BL, WB, and CL.

	Content of dietary fiber	WHC	SV
	(%)	g water/g	mL/g
BL	37.8	4.1 ± 0.2^a^	7.1 ± 0.3^a^
WB	38.1	2.9 ± 0.3^b^	3.9 ± 0.1^b^
CL	96.0	2.5 ± 0.2^b^	4.4 ± 0.2^b^

BL: young barley leaf powder, WB: wheat bran, CL: cellulose, WHC: water-holding capacity, and SV: setting volume in water.

^a,b^Data of WHC and SV, respectively, with different alphabetical letters are significantly different at *P* < 0.05.

## References

[1] Koga R, Meng T, Nakamura E (2013). The effect of photo-irradiation on the growth and ingredient composition of young green barley (Hordeum vulgare). *Agricultural Sciences*.

[2] Koga R, Meng T, Nakamura E (2013). Model examination for the effect of treading stress on young green barley (*Hordeum vulgare*). *The American Journal of Plant Sciences*.

[3] Takano A, Kamiya T, Tomozawa H (2013). Insoluble fiber in young barley leaf suppresses the increment of postprandial blood glucose level by increasing the digesta viscosity. *Evidence-Based Complementary and Alternative Medicine*.

[4] Ohtake H, Nonaka S, Sawada Y, Hagiwara Y, Hagiwara H, Kubota K (1985). Studies on the constituents of green juice from young barley leaves. Effect on dietarily induced hypercholesterolemia in rats. *Yakugaku Zasshi*.

[5] Yu YM, Wu CH, Tseng YH, Tsai CE, Chang W (2002). Antioxidative and hypolipidemic effects of barley leaf essence in a rabbit model of atherosclerosis. *Japanese Journal of Pharmacology*.

[6] Muetzel S, Hoffmann EM, Becker K (2003). Supplementation of barley straw with Sesbania pachycarpa leaves in vitro: effects on fermentation variables and rumen microbial population structure quantified by ribosomal RNA-targeted probes. *British Journal of Nutrition*.

[7] Yamaura K, Nakayama N, Shimada M, Bi Y, Fukata H, Ueno K (2012). Antidepressant-like effects of young green barley leaf (Hordeum vulgare L.) in the mouse forced swimming test. *Pharmacognosy Research*.

[8] Yu Y-M, Chang W-C, Chang C-T, Hsieh C, Tsai CE (2002). Effects of young barley leaf extract and antioxidative vitamins on LDL oxidation and free radical scavenging activities in type 2 diabetes. *Diabetes and Metabolism*.

[9] Ikeguchi M, Ariura Y, Takagaki K (2004). Effects of young barley leaf powder on fecal weight and fecal microflora in healthy women. *Journal of Japanese Association for Dietary Fiber Research*.

[10] Ikeguchi M, Kobayashi M, Ariura Y, et al (2005). Effects of young barley leaf powder on defecation frequency and fecal characteristics of healthy volunteer. *Journal of Japanese Association for Dietary Fiber Research*.

[11] Ikeguchi M, Kusaba K, Kawamura K (2006). Effects of drink containing young barley leaf powder on defecation of adult with mild constipation. *Journal of Japanese Council for Advanced Food Ingredients Research*.

[12] Eastwood MA, Kirkpatrick JR, Mitchell WD, Bone A, Hamilton T (1973). Effects of dietary supplements of wheat bran and cellulose on faeces and bowel function. *British Medical Journal*.

[13] Spiller GA, Chernoff MC, Hill RA, Gates JE, Nassar JJ, Shipley EA (1980). Effect of purified cellulose, pectin, and a low-residue diet on fecal volatile fatty acids, transit time, and fecal weight in humans. *The American Journal of Clinical Nutrition*.

[14] Slavin JL, Marlett JA (1980). Influence of refined cellulose on human bowel function and calcium and magnesium balance. *The American Journal of Clinical Nutrition*.

[15] Hillman L, Peters S, Fisher A, Pomare EW (1983). Differing effects of pectin, cellulose and lignin on stool pH, transit time and weight. *The British Journal of Nutrition*.

[16] Guerin-Deremaux L, Ringard F, Desailly F, Wils D (2010). Effects of a soluble dietary fibre NUTRIOSE on colonic fermentation and excretion rates in rats. *Nutrition Research and Practice*.

[17] Marzio L, Del Bianco R, Donne MD, Pieramico O, Cuccurullo F (1989). Mouth-to-cecum transit time in patients affected by chronic constipation: effect of glucomannan. *The American Journal of Gastroenterology*.

[18] Hayakawa T, Yamashita K, Nakano B (2003). Effects of ingestion of prune dietary fiber on cecal fermentation and fecal output in rats. *Journal of Japan Society of Nutrition and Food Science*.

[19] Takeda H, Kiriyama S (1979). Correlation between the physical properties of dietary fibers and their protective activity against amaranth toxicity in rats. *The Journal of Nutrition*.

[20] Middleton HE, Byers HG (1934). The settling volume of soils. *Soil Science*.

[21] Burkitt DP, Walker AR, Painter NS (1972). Effect of dietary fibre on stools and the transit-times, and its role in the causation of disease.. *The Lancet*.

[22] Endo K, Kumemura M, Nakamura K (1991). Effect of high cholesterol diet and polydextrose supplementation on the microflora, bacterial enzyme activity, putrefactive products, volatile fatty acid (VFA) profile, weight, and pH of the feces in healthy volunteers. *Bifidobacteria and Microflora*.

[23] Okuma K, Masuda I (2002). Indigesutable fractions of starch hydrolyzactes and their determination method. *Journal of Applied Glycoscience*.

[24] Ito M, Deguchi Y, Miyamori A (1990). Effect of administration of galactooligosaccharides on the human fecal microflora, stool weight and abdominal sensation. *Microbial Ecology in Health and Disease*.

[25] Ito M, Kimura M, Deguchi Y, Miyamori-Watabe A, Yajima T, Kan T (1993). Effects of transgalactosylated disaccharides on the human intestinal microflora and their metabolism. *Journal of Nutritional Science and Vitaminology*.

[26] Roberfroid M, Gibson GR, Hoyles L (2010). Prebiotic effects: metabolic and health benefits. *British Journal of Nutrition*.

[27] Lewis SJ, Heaton KW (1999). Roughage revisited: the effect on intestinal function of inert plastic particles of different sizes and shape. *Digestive Diseases and Sciences*.

